# An Efficient Big Data Anonymization Algorithm Based on Chaos and Perturbation Techniques †

**DOI:** 10.3390/e20050373

**Published:** 2018-05-17

**Authors:** Can Eyupoglu, Muhammed Ali Aydin, Abdul Halim Zaim, Ahmet Sertbas

**Affiliations:** 1 Department of Computer Engineering, Istanbul Commerce University, Istanbul 34840, Turkey; 2 Department of Computer Engineering, Istanbul University, Istanbul 34320, Turkey

**Keywords:** big data, chaos, data anonymization, data perturbation, privacy preserving

## Abstract

The topic of big data has attracted increasing interest in recent years. The emergence of big data leads to new difficulties in terms of protection models used for data privacy, which is of necessity for sharing and processing data. Protecting individuals’ sensitive information while maintaining the usability of the data set published is the most important challenge in privacy preserving. In this regard, data anonymization methods are utilized in order to protect data against identity disclosure and linking attacks. In this study, a novel data anonymization algorithm based on chaos and perturbation has been proposed for privacy and utility preserving in big data. The performance of the proposed algorithm is evaluated in terms of Kullback–Leibler divergence, probabilistic anonymity, classification accuracy, F-measure and execution time. The experimental results have shown that the proposed algorithm is efficient and performs better in terms of Kullback–Leibler divergence, classification accuracy and F-measure compared to most of the existing algorithms using the same data set. Resulting from applying chaos to perturb data, such successful algorithm is promising to be used in privacy preserving data mining and data publishing.

## 1. Introduction

Big data has become a hot topic in the fields of academia, scientific research, IT industry, finance and business [1,2,3]. Recently, the amount of data created in digital world has increased excessively [4]. In 2011, 1.8 zettabytes of data were generated, doubling every two years according to the research of International Data Corporation (IDC) [5]. It is anticipated that the amount of data will increase 300 times from 2005 to 2020 [6]. There are many investments conducted by health care industry, biomedical companies, advertising sector, private firms and governmental agencies in the collection, aggregation and sharing of huge amounts of personal data [7].

Big data may contain sensitive personal identifiable information that requires protection from unauthorized access and release [2,8,9]. From the point of view of security, the biggest challenge in big data is preservation of individuals’ privacy [10,11]. Guaranteeing individuals’ data privacy is mandatory when sharing private information on distributed environments [12] and the Internet of Things (IoT) [13,14,15] according to privacy laws [16]. Privacy preserving data mining [17] and privacy preserving data publishing methods [18,19] are necessary for publishing and sharing data.

In big data, modifying the original data before publishing or sharing is essential for the data owner as individuals’ private information is not to be visible in the published data set. The modification of sensitive data decreases data utility, which, on the contrary, should be convenient for sustaining the usefulness of data. This data modification process for privacy and utility of data, called as privacy preserving data publishing, protects original data sets, when releasing data. An original data set consists of four kinds of attributes. The attributes that directly identify individuals and have unique values are called identifier (ID), such as name, identity number and phone number. Sensitive attributes (SA) are the attributes that should be hidden while publishing and sharing data (e.g., salary and disease). The attributes that can be utilized by a malicious person to reveal an individual’s identity are called quasi-identifier (QI), including age and sex. Other attributes are non-sensitive attributes (NSA). Before publishing, the original data set is anonymized by deleting identifiers and modifying quasi-identifiers, thereby preserving individuals’ privacy [20].

In order to preserve privacy, there are five types of anonymization operations, namely generalization, suppression, anatomization, permutation and perturbation. Generalization replaces values with more generic ones. Suppression removes specific values from data sets (e.g., replacing values with specific ones like “*”). Anatomization disassociates relations between quasi-identifiers and sensitive attributes. Furthermore, permutation disassociates a relation between a quasi-identifier and sensitive attribute by dividing a number of data records into groups and mixing their sensitive values in every group. Perturbation replaces original values with new ones by interchanging, adding noise or creating synthetic data. These anonymization operations decrease data utility, which is represented by information loss in general. In other words, higher data utility means lower information loss [18,20].

Various studies utilizing the aforementioned operations have been done by now. In this paper, to address the problems of data utility and information loss, a new anonymization algorithm using chaos and perturbation operation is introduced. Our main contribution is developing a comprehensive privacy preserving data publishing algorithm which is independent of data set type and can be applied on both numerical and categorical attributes. The proposed algorithm has higher data utility due to analyzing frequency of unique attribute values for every quasi-identifier, determining crucial values in compliance with frequency analysis and performing perturbation operation only for these determined crucial values. Another significant contribution of this study is to prove the efficiency of chaos, an interdisciplinary theory commonly used for randomness of systems, in perturbing data. To the best of the authors’ knowledge, there is no other work in the literature pertaining to the utility of chaos in privacy preserving of big data in this framework. Great success of chaos in randomization motivated the authors to explore its utility in data perturbation. Evaluating the performance of the proposed algorithm through different metrics, the test results demonstrate that the algorithm is effective compared to previous studies.

The organization of the rest of the paper is as follows: in Section 2, the related works are given. Section 3 introduces the proposed privacy preserving algorithm. In Section 4, privacy analyses and experimental results of the proposed algorithms are demonstrated comparing with the existing algorithms. Finally, conclusions being under study are summarized in Section 5.

## 2. Related Works

In privacy preserving data mining and data publishing, protection of privacy is achieved using various methods such as data anonymization [16,21,22,23,24,25,26,27], data perturbation [28,29,30,31,32,33,34], data randomization [35,36,37,38] and cryptography [39,40], among which *k*-anonymity and *k*-anonymity based algorithms like Datafly [23], Incognito [41] and Mondrian [42] are the most commonly used techniques. *k*-anonymization is the process whereby the values of quasi-identifiers are modified so that any individual in the anonymized data set is indistinguishable from at least *k* − 1 other ones [20]. Table 1 shows a sample original data set where age, sex and ZIP code™ (postal code) are the quasi-identifiers and disease is the sensitive attribute.

The two-anonymous form of this original data set obtained by utilizing *k*-anonymization is demonstrated in Table 2. As seen from the table, using generalization and suppression operations, five equivalence classes having the same values are attained. These 2-anonymous groups tackle with identity disclosure and linking attacks.

Machanavajjhala et al. [43] introduced the *l*-diversity principle in order to improve *k*-anonymity in which sensitive attributes lack diversity. *l*-diversity focuses on the relations between quasi-identifiers and sensitive attributes. If a quasi-identifier group includes at least *l* well-represented sensitive attribute values, it satisfies *l*-diversity. Furthermore, entropy *l*-diversity is satisfied if the entropy of sensitive attribute is bigger than ln *l* for every quasi-identifier group in a data set. In order to overcome the limitations of the *l*-diversity principle, Li et al. [44] proposed the *t*-closeness principle coping with attribute disclosure and similarity attack. Sun et al. [45] offered a top-down anonymization model by improving *l*-diversity and entropy *l*-diversity.

Agrawal and Srikant [46] presented a value distortion method to preserve privacy via adding random noise from a Gaussian distribution to original data set. This method was improved by Agrawal and Aggarwal [47] to create a better distribution.

Evfimievski et al. [48] proposed an association rule mining framework by randomizing data, which was then modified by Evfimievski et al. [49] to restrict privacy breaches without data distribution information. Furthermore, Rizvi and Haritsa [50] presented a probabilistic distortion based scheme to ensure privacy.

Yang and Qiao [33] presented an anonymization method breaking randomly the links between quasi-identifiers and sensitive attribute for privacy protection and knowledge preservation. Chen et al. [28] proposed a data perturbation method combining reversible data hiding and difference hiding to solve the knowledge and data distortion problem in privacy preserving data mining.

Dwork [51] proposed differential privacy which has been widely used to resist background knowledge attacks in privacy preserving data publishing [52,53]. Differential privacy approach is protecting privacy via adding noise to the values correlated to the confidential data in the area of privacy preserving statistical databases including individual records and aiming the support of information discovery [54]. The Laplace mechanism [55] adding random noise sampled from the Laplace distribution into the record counts is the most commonly used approach to provide differential privacy [56]. Besides, McSherry and Talwar [57] presented an exponential mechanism ensuring the output quality to achieve differential privacy.

Mohammed et al. [58] introduced the first generalization-based privacy preserving data publishing algorithm guaranteeing differential privacy and protecting information for further classification analysis. Chen et al. [59] proposed the first trajectory data publishing approach with the requirements of differential privacy. Li et al. [60] presented a *k*-anonymization technique utilizing suppression and sampling operations in order to satisfy differential privacy. Soria-Comas et al. [61] proposed a microaggregation-based *k*-anonymity approach combining *k*-anonymity and differential privacy to enhance data utility. Fouad et al. [62] introduced a differential privacy preserving algorithm based on supermodularity and random sampling. Wang and Jin [63] proposed a differential privacy multidimensional data publishing model adapted from *kd*-tree algorithm [64]. Zaman et al. [65] presented a 2-layer differential privacy preserving technique using generalization operation and the Laplace mechanism for data sanitization. Koufogiannis and Pappas [66] introduced a privacy preserving mechanism based on differential privacy for the protection of dynamical systems. Li et al. [67] proposed an insensitive clustering algorithm for differential privacy data protection and publishing.

Dong et al. [68] presented two effective privacy preserving data deduplication techniques for data cleaning as a service (DCaS) enabling corporations to outsource their data sets and data cleaning demands to third-party service providers. These techniques resist frequency analysis and known-scheme attacks.

In a recent study, Nayahi and Kavitha [21] proposed a (G, S) clustering algorithm that is resilient to similarity attack for anonymizing data and preserving sensitive attributes. Afterwards, they modified their (G, S) clustering algorithm and proposed the KNN-(G, S) clustering algorithm [16] using the *k*-Nearest Neighbours technique (*k*-NN) to protect sensitive data against probabilistic inference attack, linking attack, homogeneity attack and similarity attack. Unlike the aforementioned methods, in this work, a new chaos and perturbation based anonymization algorithm has been proposed to protect privacy and utility in big data.

## 3. Proposed Privacy Preserving Algorithm 

**Figure 1:**
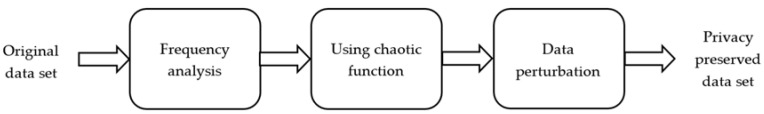
General block diagram of the proposed algorithm.

In this study, privacy and utility preservation are achieved using chaos and data perturbation techniques. The general block diagram of the proposed algorithm consists of the three main stages illustrated in Figure 1. The first stage is for analyzing the frequency of unique attribute values for each quasi-identifier and then finding the crucial values according to frequency analysis. The second stage utilizes a chaotic function to designate new values for the chosen crucial values. In the final stage, data perturbation is performed.

An overview of the proposed algorithm is presented in Algorithm 1, which consists of these eight steps:

Step 1: The original input data set *D*, quasi-identifier attributes *QI* (*QI*_1_*, QI*_2_*, …, QI_q_*), and sensitive attribute *SA* are specified.

Step 2: The unique attribute values for each *QI* are found. |*D*| is the size of input data set *D* and |*QI*| is the number of quasi-identifier attributes *QI*.

Step 3: The number of records containing the unique attribute values is computed for each *QI*.

Step 4: The unique attribute values are sorted in ascending order in accordance with the frequency.

Step 5: The record places of the unique attribute values in *D* are found for subsequent randomization and replacement processes.

Step 6: The number of crucial unique attribute values is calculated for each *QI* using Equation (1):

(1) $${r\;=\;\mathrm{round}\;(\log}_{2}\mathrm{number}\;\mathrm{of}\;\mathrm{unique}\;\mathrm{attribute}\;\mathrm{values})$$

The less the number of unique attribute values for a particular *QI*, the more crucial for identity disclosure and linking attacks. These attributes might be utilized by an intruder to infer the sensitive attribute of an individual.

**Figure 2:**
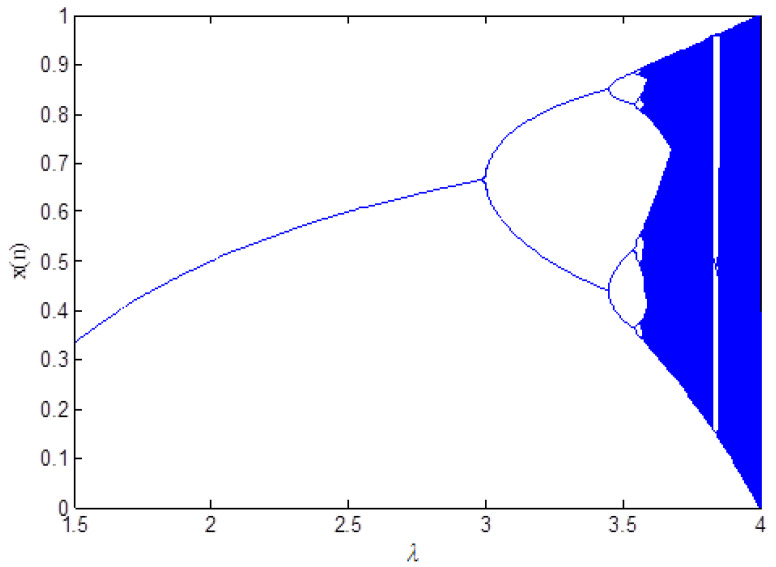
Bifurcation diagram of logistic map.

Step 7: The new attribute values for the selected crucial unique values are determined using a chaotic function known as logistic map (Equation (2)):

(2) f(x) = λx(1 – x)

where 3.57 < *λ* < 4. The chaotic behaviour of the function depends completely on *λ* value. In order to make the function operate in the most chaotic region, *λ* is defined in the range of 3.99 and 4 [69]. Figure 2 shows the bifurcation diagram of the logistic map. As it is seen from Figure 2, the function output takes on different values in the range of 0 and 1 when *λ* value approaches to 4. The aim of using logistic map in this study is to take advantage of its familiar chaotic behaviour in order for data perturbation.

Step 8: The selected record values in *D* are replaced with the determined new attribute values. Finally, the privacy preserved data set *D_p_* is obtained.

**Figure 3:**
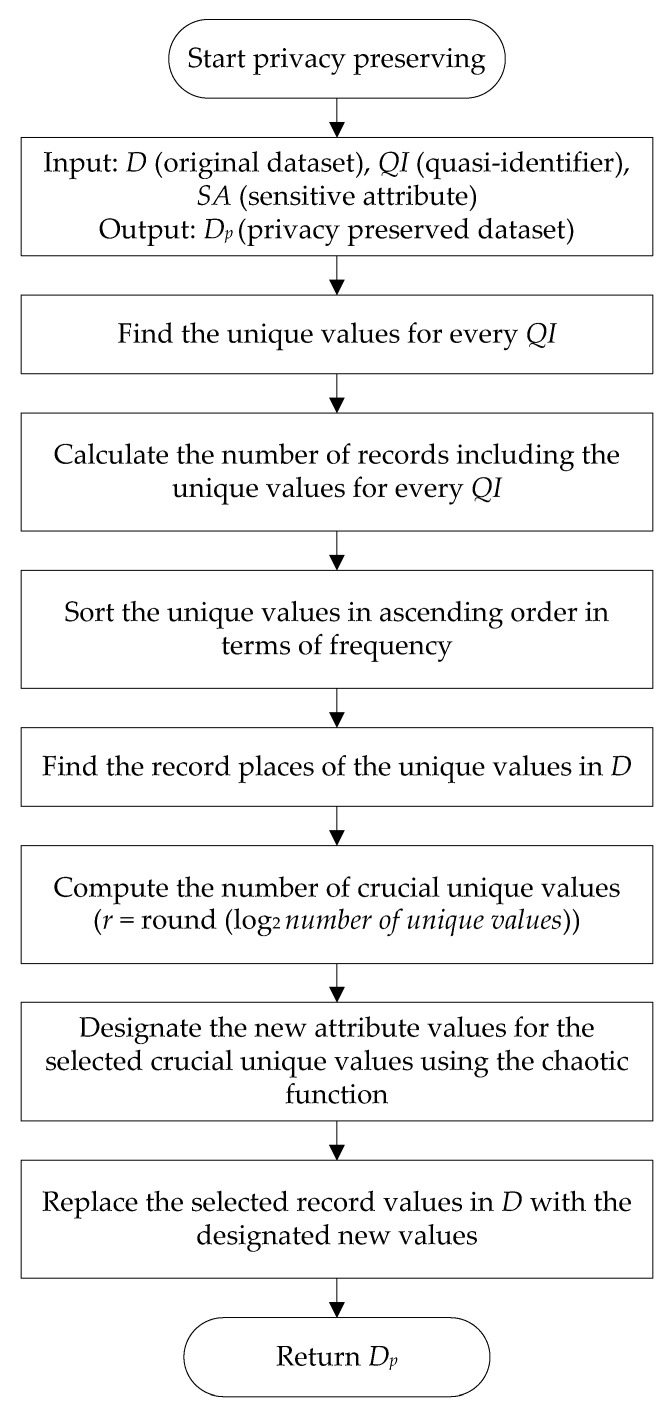
Operational flowchart of the proposed algorithm.

The flowchart of the privacy preserving process is demonstrated in Figure 3 to better clarify the algorithm.

**Algorithm 1:** Efficient Privacy Preserving Algorithm**Input:** Original input data set *D*, quasi-identifier attributes *QI (QI*_1_, *QI*_2_, …, *QI_q_)*, and sensitive attribute *SA*
**Output:** Privacy preserved data set *D_p_*
**Initial assignments:**
*c* = 0, *λ* = 3.99, *iteration* = 400
1:*d* = |*D*|2:*q* = |*QI*|3:**for***i* = 1 to *q* **do**4:  *nu_i_* = number of unique values for each *QI_i_*5:  **for** *j* = 1 to *nu_i_* **do**6:    *u_ij_* = unique values for each *QI_i_*7:    *v_ij_* = number of records containing the unique value *u_ij_*8:  **end for**9:
**end for**
10:Sort *u_ij_* in ascending order based on *v_ij_* for each *QI_i_*11:*record_place_i_* = Ø (the size *d* × *nu_i_* for each *QI_i_*)12:**for***i* = 1 to *q* **do**13:  **for** *j* = 1 to *nu_i_* **do**14:    **for** *k* = 1 to *d* **do**15:      **if** *k*-th record value in *QI_i_* == *j*-th value in sorted *u_ij_* **then**16:        *c*++17:        *record_place_i_* (*c*, *j*) = *j*18:      **else**19:        continue20:      **end if**21:    **end for**22:    *c* = 023:  **end for**24:
**end for**
25:**for***i* = 1 to *q* **do**26:  *r_i_* = round (log_2_ *nu_i_*)27:
**end for**
28:**for***i* = 1 to *q* **do**29:  *x_i1_* = 0.130:  **for***j* = 1 to *iteration* **do**31:    *x_ij + 1_* = *λ* × *x_ij_* × (1 − *x_ij_*)32:  **end for**33:
**end for**
34:Determine the new attribute values for the first *r_i_* value in sorted unique values *u_ij_* based on the record places *x_ij_* for each *QI_i_*35:Replace the chosen record values in *D* with the determined new values36:Return *D_p_*


## 4. Privacy Analyses and Experimental Results

In this section, the performance metrics used for evaluation of the proposed privacy preserving algorithm are presented. These metrics are Kullback–Leibler divergence (relative entropy), probabilistic anonymity, classification accuracy, F-measure and execution time. The proposed algorithm is implemented in MATLAB R2016a (MathWorks, Natick, MA, USA) running on the Windows 7 64-bit operating system on a personal computer equipped with 16 GB RAM and an Intel Core i7-3820 (3.60 GHz) processor. The classification accuracy and F-measure results of the proposed algorithm are obtained using various classification techniques provided in Weka 3.8 (University of Waikato, Hamilton, Waikato, New Zealand).

### 4.1. Data Set Description

The performance of the proposed algorithm is evaluated on the Adult data set extracted from the 1994 U.S. Census database [70]. The reason why this data set is used in this study is that it is utilized as a benchmark for privacy analysis of algorithms in the literature. Besides, the data set is available online from the Machine Learning Repository at the University of California-Irvine [71]. It contains 32,561 records and the total number of records without missing values is 30,162. The number of attributes is 15 (six continuous and nine nominal). In the data set, 7508 instances are in class “>50 K” and 22,654 instances are in class “≤50 K”. The detailed description of the Adult data set is shown in Table 3.

To demonstrate the scalability of the proposed algorithm on big data, the Adult data set is uniformly enlarged as four data sets which have ~60 K, 120 K, 240 K and 480 K records, respectively. Furthermore, data doubling is performed evenly without corrupting data integrity to evaluate the classification accuracy, F-measure and execution time performance of the proposed algorithm on *k*-anonymous forms of the Adult data set, ensuring *k* = 2, 4, 8 and 16. In order for comparing the performance of the proposed algorithm with the existing algorithms, three attributes are selected as quasi-identifiers which are “age”, “race” and “sex”. Moreover, the attribute “income” is chosen as the sensitive attribute (class attribute).

### 4.2. Kullback–Leibler Divergence

Kullback–Leibler divergence (KL divergence) is used to quantify the difference between two distributions [45,72]. In privacy preserving, it is utilized for computing the distance between original and privacy preserved data sets. The KL divergence metric is defined as:

(3) $$\mathrm{KL}\;\mathrm{divergence}\;=\;\underset{x}{\sum }p(x)\log\frac{p(x)}{q(x)}$$

where *p*(*x*) and *q*(*x*) are two distributions [21]. The KL divergence is non-negative and it is 0 if the two distributions are the same [44]. In this study, *p*(*x*) and *q*(*x*) distributions are used for privacy preserved and original data sets, respectively.

**Figure 4:**
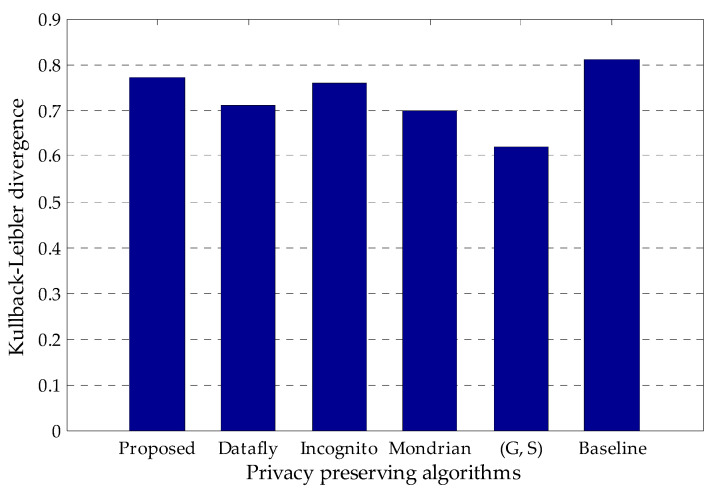
Comparison of Kullback–Leibler divergence (KL divergence) of the proposed algorithm with the existing methods.

Figure 4 presents the comparison of KL divergence of the proposed algorithm with the existing methods which are Datafly, Incognito, Mondrian and (G, S). The baseline value is the entropy of the sensitive attribute in the original Adult data set. As can be seen from the figure, KL divergence of the proposed algorithm is better than the existing algorithms and very close to the baseline value. This result shows that the proposed algorithm slightly distorts the original data set. In addition, it has higher data utility, resulting from performing perturbation operation only for the specified crucial values with regard to the frequency analysis of unique attribute values for each quasi-identifier.

### 4.3. Probabilistic Anonymity

Probabilistic anonymity is a statistical measurement for privacy or anonymity defined and proved by [33]. In a privacy preserved data set, the attacker cannot infer the original relations from the corresponding relations. The probabilistic anonymity measures the inability for inference.

**Definition** **1** (probabilistic anonymity). *Given a data set D and its anonymized form D’. Let r be a record in D and r’ ∈ D’ be its anonymized version. Symbolize r(QI) as the value combination of the quasi-identifier in r. The probabilistic anonymity of D’ is defined as 1/P(r(QI)|r’(QI)). P(r(QI)|r’(QI)) is the probability that r(QI) might be inferred given r’(QI). Let Q_i_, i = 1, …, m be the i-th quasi-identifier attribute in D and Entropy(Q_i_) be the entropy value of Q_i_. The probabilistic anonymity of D’ is denoted as Pa(D’) and defined as*:

(4) $${\mathrm{Pa}(D^{\prime })\;=\;e}^{\mathrm{Entropy}(Q_{i})}$$

*Pa(D’) attains the maximal value when*:

(5) $$p_{i}\;=\;\frac{e^{\mathrm{Entropy}(Q_{i})}}{\sum_{j=1}^{m}e^{\mathrm{Entropy}(Q_{i})}}$$

This proposition can be used as a general measurement for computing the probabilistic anonymity. An estimation of the scaled *Pa*(*D*’) can be made by calculating the geometric mean of all quasi-identifier diversities when:

(6) $$p_{i}\;=\;\frac{1}{m},\;i\;=\;1,\ldots ,\;m$$

(7) $$\ln\;\mathrm{Pa}(D^{\prime })\;=\;\ln m+\overset{m}{\underset{i=1}{\sum }}(\frac{1}{m}\mathrm{Entropy}(Q_{i}))=\ln(m(\overset{m}{\underset{i=1}{\prod }}{\mathrm{Diversity}}_{i}{)}^{\frac{1}{m}})$$

where:

(8) $${\mathrm{Diversity}}_{i}{\;=\;e}^{\mathrm{Entropy}(Q_{i})}$$

The probability of estimating the original value of a quasi-identifier for an arbitrary record in *D* is calculated as 1/*Pa*(*D*’). Furthermore, this probability shows the confidence of a user for associating a sensitive value with an individual. Derived from Equation (7), *Pa*(*D*’) is mostly greater than the geometric mean of all quasi-identifier diversities. In a similar way, *Pa*(*D*’) is mostly greater than the sensitive attribute diversity. Given a diversity of a sensitive attribute *Diversity_s_*. The maximal confidence of a user in inferring the corresponding sensitivity is 1/*Diversity_s_* when it is certain that an individual is in the data set. Readers are referred to [33] for proof and further details.

The probabilistic anonymity of the proposed algorithm for the Adult data set is calculated using Equation (7) for which the corresponding value is 24.53. For an arbitrary record in the Adult data set, the estimation probability for the original value of a quasi-identifier is 0.04. These results show that the probabilistic anonymity of the proposed algorithm is quite good.

### 4.4. Classification Accuracy

The classification accuracy is the percentage of correctly classified test set tuples and defined as:

(9) $$\mathrm{Classification}\;\mathrm{accuracy}\;=\;\frac{\mathrm{TP}\;+\;\mathrm{TN}}{P\;+\;N}$$

**Figure 5:**
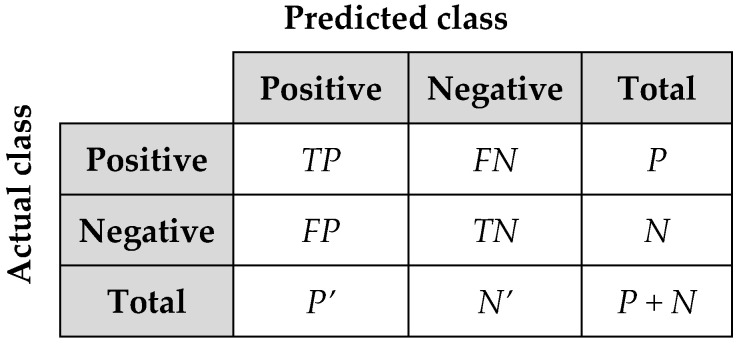
Confusion matrix.

*P* is the number of positive tuples. *N* is the number of negative tuples. True positives (*TP*) are correctly labelled as positive tuples. True negatives (*TN*) are correctly labelled as negative tuples. False positives (*FP*) are the negative tuples which are mislabelled as positive. False negatives (*FN*) are the positive tuples which are incorrectly labelled as negative. *P*’ is the number of labelled positive tuples, and *N’* is the number of labelled negative tuples [73]. Figure 5 shows the confusion matrix that is the summary of these terms.

The classification accuracy of the proposed method is investigated using four different classifiers, which are Voted Perceptron (VP), OneR, Naive Bayes (NB) and Decision Tree (J48). For *k*-fold cross validation technique, the results of the classification accuracy of the proposed algorithm for five data sets with different sizes are demonstrated in Table 4. 2-fold, 5-fold and 10-fold cross validation are performed for each classifier. The classification accuracies of the original and privacy preserved forms of the data sets on which the proposed algorithm are applied are compared with each other to evaluate the proposed algorithm. Higher values of classification accuracy are preferred and classification accuracy values which are closer to the original values mean that the information loss is low, referring to higher data utility.

As seen from Table 4, a rise in *k* value causes a small increase in classification accuracy for each data set in general. For all data sets, classification accuracies of privacy preserved data sets are the same or very close to the originals. The classification accuracies of the original and privacy preserved data sets are the same for Voted Perceptron and OneR classifiers and almost equal for Naive Bayes and J48 classifiers. Besides, the best accuracy values are achieved using J48 classifier for each data set.

For the same data set, quasi-identifiers, sensitive attribute, and classification algorithms, the comparison of classification accuracy of the proposed algorithm with the existing methods, namely Datafly, Incognito, Mondrian, Entropy *l*-diversity, (G, S) and KNN-(G, S) in 10-fold cross validation scheme is shown in Table 5.

**Figure 6:**
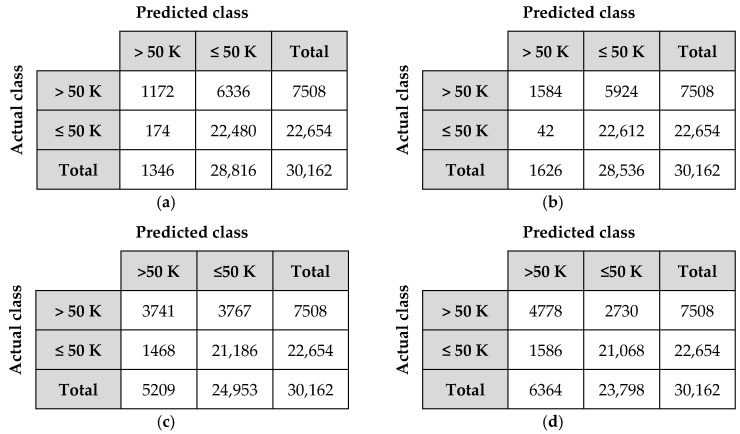
Confusion matrices: (**a**) Voted Perceptron; (**b**) OneR; (**c**) Naive Bayes; (**d**) J48.

It can be seen from the table that the classification accuracy of the proposed algorithm is better than the existing algorithms in all cases of Voted Perceptron, OneR and J48 classifiers. The performance of the proposed privacy preserving algorithm is the same with the original Adult data set in Voted Perceptron and OneR classifiers. Furthermore, the classification accuracy of the proposed algorithm is almost the same with the original value in J48 classifier. J48 classifier also gives the best accuracy results for all algorithms. Besides, the confusion matrices of the proposed algorithm pertaining to Voted Perceptron, OneR, Naive Bayes and J48 for the Adult data set in 10-fold cross validation scheme are demonstrated in Figure 6.

### 4.5. F-Measure

The F-measure also known as F-score and F_1_ score is a measure for accuracy of a test and utilized in order for evaluating classification techniques. The F-measure is defined as:

(10) $$F-\mathrm{measure}\;=\;\frac{2\;\times \;\mathrm{precision}\;\times \;\mathrm{recall}}{\mathrm{precision}\;+\;\mathrm{recall}}$$

where *precision* and *recall* are the measures of exactness and completeness, respectively. These measures are calculated as [73]:

(11) $$\mathrm{Precision}\;=\;\frac{\mathrm{TP}}{\mathrm{TP}\;+\;\mathrm{FP}}$$

(12) $$\mathrm{Recall}\;=\;\frac{\mathrm{TP}}{\mathrm{TP}\;+\;\mathrm{FN}}\;=\;\frac{\mathrm{TP}}{P}$$

To analyse the F-measure performance of the proposed algorithm, four classification algorithms are utilized. The results of the F-measure of the proposed algorithm for five data sets with different sizes are shown in Table 6 for *k*-fold cross validation technique. For each classification algorithm, 2-fold, 5-fold and 10-fold cross validation are carried out. In order to measure the performance of the proposed algorithm, F-measures of the original and privacy preserved versions of the data sets are compared with each other. Higher values of F-measure are preferred and closer F-measure values to the originals are better.

It can be seen from the analysis of Table 6 that F-measure values rise slightly with an increase in k value for each data set in general. The proposed algorithm achieves the best F-measure values with J48 classification technique compared to Voted Perceptron, OneR and Naive Bayes. F-measure of privacy preserved data sets are the same or very close to the original values for all data sets. For Voted Perceptron and OneR classifiers, F-measures of the original and privacy preserved data sets are the same and almost equal for Naive Bayes and J48 classifiers.

The F-measure comparison of the proposed algorithm with the existing methods for the same experiment conditions in 10-fold cross validation scheme is demonstrated in Table 7. As seen from the table, the proposed algorithm shows better or equal performance in all cases of Voted Perceptron and OneR classification algorithms compared to the existing algorithms. In J48 classifier, the performance of the proposed algorithm is better than all existing algorithms and the same with the original Adult data set. F-measure of the proposed algorithm is very close to the original in Naive Bayes classifier. Besides, the J48 classifier is better than other three classifiers in terms of F-measure for all algorithms.

### 4.6. Execution Time

**Figure 7:**
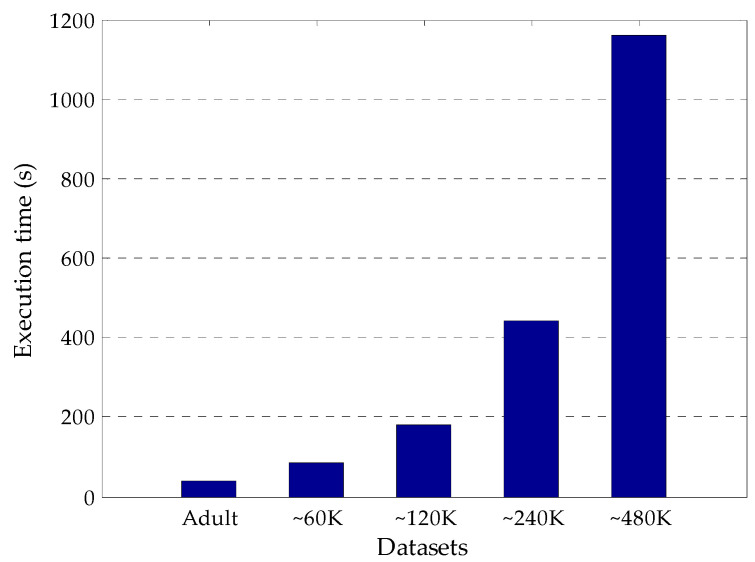
Execution time performance of the proposed algorithm for various data sets.

In this study, five data sets with different sizes are used to show the feasibility and scalability of the proposed algorithm on big data. The execution time performance of the proposed algorithm is investigated utilizing the Adult data set and its four enlarged versions including ~60 K, 120 K, 240 K and 480 K records (Figure 7). As seen from the figure, as the number of records in the data sets increases, the execution time of the proposed algorithm rises. Furthermore, the results of execution time for each data set indicate that the proposed algorithm is optimal in terms of feasibility and scalability.

## 5. Conclusions

In this paper, a new chaos and perturbation based algorithm is introduced for privacy and utility preserving in big data. The scalability and feasibility of the proposed algorithm are evaluated using several data sets with different sizes. Kullback–Leibler divergence, probabilistic anonymity, classification accuracy, F-measure and execution time are utilized as evaluation metrics. Privacy analyses and experimental results demonstrate that the proposed algorithm performs better than the previous studies with regards to Kullback–Leibler divergence, classification accuracy and F-measure in the same experiment conditions. Probabilistic anonymity and execution time performance of the proposed algorithm are sufficient. Taking into consideration the success of the proposed algorithm which results from utilizing a chaotic function for data perturbation purpose, the algorithm ensures its suitability for the protection of individuals’ privacy before publishing and sharing data.

## Figures and Tables

**Table 1:** A sample original data set.

Age	Sex	ZIP Code™	Disease
32	Female	34200	Breast Cancer
38	Female	34800	Kidney Cancer
64	Male	40008	Skin Cancer
69	Female	40001	Bone Cancer
53	Male	65330	Skin Cancer
56	Male	65380	Kidney Cancer
75	Female	20005	Breast Cancer
76	Male	20009	Prostate Cancer
41	Male	85000	Lung Cancer
47	Male	87000	Lung Cancer

**Table 2:** 2-anonymous form of original data set in Table 1.

Age	Sex	ZIP Code™	Disease
[30–40]	Female	34 ***	Breast Cancer
[30–40]	Female	34 ***	Kidney Cancer
[60–70]	*	4000 *	Skin Cancer
[60–70]	*	4000 *	Bone Cancer
[50–60]	Male	653 **	Skin Cancer
[50–60]	Male	653 **	Kidney Cancer
[70–80]	*	2000 *	Breast Cancer
[70–80]	*	2000 *	Prostate Cancer
[40–50]	Male	8 ****	Lung Cancer
[40–50]	Male	8 ****	Lung Cancer

**Table 3:** Detailed description of the Adult data set.

Attribute	Attribute Type	Domain
age	continuous	[17–90]
workclass	nominal	Private, Self-emp-not-inc, Self-emp-inc, Federal-gov, Local-gov, State-gov, Without-pay, Never-worked
fnlwgt	continuous	[19, 214–1, 226, 583]
education	nominal	Bachelors, Some-college, 11th, HS-grad, Prof-school, Assoc-acdm, Assoc-voc, 9th, 7th–8th, 12th, Masters, 1st–4th, 10th, Doctorate, 5th–6th, Preschool
education-num	continuous	[1–16]
marital-status	nominal	Married-civ-spouse, Divorced, Never-married, Separated, Widowed, Married-spouse-absent, Married-AF-spouse
occupation	nominal	Tech-support, Craft-repair, Other-service, Sales, Exec-managerial, Prof-specialty, Handlers-cleaners, Machine-op-inspct, Adm-clerical, Farming-fishing, Transport-moving, Priv-house-serv, Protective-serv, Armed-Forces
relationship	nominal	Wife, Own-child, Husband, Not-in-family, Other-relative, Unmarried
race	nominal	White, Asian-Pac-Islander, Amer-Indian-Eskimo, Other, Black
sex	nominal	Female, Male
capital-gain	continuous	[0–99,999]
capital-loss	continuous	[0–4356]
hours-per-week	continuous	[1–99]
native-country	nominal	United-States, Cambodia, England, Puerto-Rico, Canada, Germany, Outlying-US (Guam-USVI-etc.), India, Japan, Greece, South, China, Cuba, Iran, Honduras, Philippines, Italy, Poland, Jamaica, Vietnam, Mexico, Portugal, Ireland, France, Dominican-Republic, Laos, Ecuador, Taiwan, Haiti, Columbia, Hungary, Guatemala, Nicaragua, Scotland, Thailand, Yugoslavia, El-Salvador, Trinadad & Tobago, Peru, Hong, Holand-The Netherlands
income (class att.)	nominal	“>50 K” and “≤50 K”

**Table 4:** Classification accuracy results of the proposed algorithm for various data sets.

Data Sets		2-Fold Cross Validation				5-Fold Cross Validation				10-Fold Cross Validation			
		VP	OneR	NB	J48	VP	OneR	NB	J48	VP	OneR	NB	J48
Adult	Original	77.84	80.21	82.75	85.03	78.36	80.21	82.84	85.71	78.42	80.22	82.88	85.73
	Privacy Preserved	77.84	80.21	82.55	85.14	78.36	80.21	82.59	85.54	78.42	80.22	82.64	85.69
~60 K	Original	78.41	80.24	82.78	87.19	78.44	75.45	82.90	88.94	78.43	75.54	82.87	89.43
	Privacy Preserved	78.41	80.24	82.58	86.92	78.44	75.45	82.65	88.73	78.43	75.54	82.64	89.31
~120 K	Original	78.45	78.16	82.83	92.15	78.46	81.20	82.88	96.95	78.45	82.31	82.89	98.13
	Privacy Preserved	78.45	78.16	82.62	92.31	78.46	81.20	82.66	96.86	78.45	82.31	82.65	98.18
~240 K	Original	78.47	83.24	82.87	98.41	78.43	86.04	82.90	99.84	78.44	87.09	82.90	99.89
	Privacy Preserved	78.47	83.24	82.65	98.39	78.43	86.04	82.65	99.83	78.44	87.09	82.65	99.89
~480 K	Original	78.40	88.69	82.90	99.86	78.42	89.30	82.90	99.98	78.44	88.73	82.90	99.99
	Privacy Preserved	78.40	88.69	82.66	99.85	78.42	89.30	82.66	99.98	78.44	88.73	82.66	99.99

**Table 5:** Comparison of classification accuracy of the proposed algorithm with the existing methods.

Privacy Preserving Algorithms	*k*	Classification Algorithms			
		VP	OneR	NB	J48
Original Adult data set	–	78.42	80.22	82.88	85.73
Datafly [23]	5	78.36	80.18	82.85	85.35
Incognito [41]	5	78.38	80.17	82.75	85.30
Mondrian [42]	5	78.38	80.17	82.83	85.00
Entropy *l*-diversity (*l* = 2) [43]	5	78.38	80.17	82.40	85.42
(G, S) [21]	5	78.43	80.21	83.46	85.16
KNN-(G, S) [16]	5	78.38	80.16	82.72	85.26
Datafly [23]	10	78.38	80.18	82.85	85.35
Incognito [41]	10	78.38	80.15	82.44	85.30
Mondrian [42]	10	78.38	80.17	82.83	84.97
Entropy *l*-diversity (*l* = 2) [43]	10	78.37	80.18	82.40	85.40
(G, S) [21]	10	78.43	80.21	83.46	85.16
KNN-(G, S) [16]	10	78.38	80.16	83.72	85.26
Datafly [23]	25	78.38	80.18	82.85	85.38
Incognito [41]	25	78.38	80.17	82.71	85.31
Mondrian [42]	25	78.38	80.17	82.84	84.99
Entropy *l*-diversity (*l* = 2) [43]	25	78.38	80.17	82.40	85.42
(G, S) [21]	25	78.44	80.20	82.12	85.16
KNN-(G, S) [16]	25	78.39	80.19	83.01	85.40
Datafly [23]	50	78.38	80.17	83.11	85.37
Incognito [41]	50	78.38	80.17	82.71	85.31
Mondrian [42]	50	78.38	80.17	82.85	85.05
Entropy *l*-diversity (*l* = 2) [43]	50	78.38	80.17	82.40	84.42
(G, S) [21]	50	78.42	80.17	83.44	85.35
KNN-(G, S) [16]	50	78.39	80.11	83.50	85.69
Proposed Algorithm	–	78.42	80.22	82.64	85.69

**Table 6:** F-measure results of the proposed algorithm for different data sets.

Data Sets		2-Fold Cross Validation				5-Fold Cross Validation				10-Fold Cross Validation			
		VP	OneR	NB	J48	VP	OneR	NB	J48	VP	OneR	NB	J48
Adult	Original	0.709	0.750	0.817	0.845	0.721	0.750	0.818	0.853	0.722	0.750	0.819	0.853
	Privacy Preserved	0.709	0.750	0.814	0.845	0.721	0.750	0.814	0.851	0.722	0.750	0.815	0.853
~60 K	Original	0.723	0.750	0.818	0.869	0.723	0.729	0.819	0.887	0.723	0.731	0.819	0.892
	Privacy Preserved	0.723	0.750	0.814	0.866	0.723	0.729	0.815	0.885	0.723	0.731	0.815	0.891
~120 K	Original	0.724	0.765	0.818	0.920	0.724	0.803	0.819	0.969	0.723	0.816	0.819	0.981
	Privacy Preserved	0.724	0.765	0.815	0.922	0.724	0.803	0.815	0.968	0.723	0.816	0.815	0.982
~240 K	Original	0.724	0.825	0.819	0.984	0.723	0.858	0.819	0.998	0.723	0.870	0.819	0.999
	Privacy Preserved	0.724	0.825	0.815	0.984	0.723	0.858	0.815	0.998	0.723	0.870	0.815	0.999
~480 K	Original	0.722	0.886	0.819	0.999	0.723	0.893	0.819	1.000	0.723	0.887	0.819	1.000
	Privacy Preserved	0.722	0.886	0.815	0.998	0.723	0.893	0.815	1.000	0.723	0.887	0.815	1.000

**Table 7:** Comparison of F-measure of the proposed algorithm with the existing methods.

Privacy Preserving Algorithms	*k*	Classification Algorithms			
		VP	OneR	NB	J48
Original Adult data set	–	0.722	0.750	0.819	0.853
Datafly [23]	5	0.722	0.750	0.819	0.850
Incognito [41]	5	0.722	0.749	0.818	0.847
Mondrian [42]	5	0.722	0.749	0.818	0.843
Entropy *l*-diversity (*l* = 2) [43]	5	0.722	0.749	0.808	0.849
(G, S) [21]	5	0.723	0.750	0.829	0.845
KNN-(G, S) [16]	5	0.722	0.749	0.817	0.847
Datafly [23]	10	0.722	0.749	0.819	0.849
Incognito [41]	10	0.722	0.749	0.812	0.848
Mondrian [42]	10	0.722	0.749	0.818	0.840
Entropy *l*-diversity (*l* = 2) [43]	10	0.722	0.750	0.808	0.849
(G, S) [21]	10	0.723	0.750	0.829	0.845
KNN-(G, S) [16]	10	0.722	0.749	0.817	0.847
Datafly [23]	25	0.722	0.749	0.819	0.849
Incognito [41]	25	0.722	0.749	0.817	0.847
Mondrian [42]	25	0.722	0.749	0.818	0.840
Entropy *l*-diversity (*l* = 2) [43]	25	0.722	0.749	0.808	0.849
(G, S) [21]	25	0.723	0.750	0.808	0.845
KNN-(G, S) [16]	25	0.722	0.749	0.822	0.849
Datafly [23]	50	0.722	0.749	0.825	0.848
Incognito [41]	50	0.722	0.749	0.817	0.847
Mondrian [42]	50	0.722	0.749	0.818	0.842
Entropy *l*-diversity (*l* = 2) [43]	50	0.722	0.749	0.808	0.849
(G, S) [21]	50	0.723	0.749	0.830	0.848
KNN-(G, S) [16]	50	0.722	0.749	0.836	0.853
Proposed Algorithm	–	0.722	0.750	0.815	0.853
